# TiO_2_ Nanorods and Pt Nanoparticles under a UV-LED for an NO_2_ Gas Sensor at Room Temperature

**DOI:** 10.3390/s21051826

**Published:** 2021-03-05

**Authors:** Jinhong Noh, Soon-Hwan Kwon, Sunghoon Park, Kyoung-Kook Kim, Yong-Jin Yoon

**Affiliations:** 1 Department of Mechanical Engineering, Korea Advanced Institute of Science and Technology, Daejeon 34141, Korea; jinhongnoh@kaist.ac.kr; 2 Department of Advanced Convergence Technology, Research Institute of Advanced Convergence Technology, Korea Polytechnic University, Siheung-si 15073, Korea; canwkd21@kpu.ac.kr; 3 Department of Intelligent and Mechatronics Engineering, Sejong University, Seoul 05006, Korea; s.park@sejong.ac.kr; 4 Department of Nano & Semiconductor Engineering, Korea Polytechnic University, Siheung-si 15073, Korea; 5 School of Mechanical and Aerospace Engineering, Nanyang Technological University, Singapore 639798, Singapore

**Keywords:** gas sensor, room temperature, photoelectrochemical performance, plasmonic effect, electron trap, TiO_2_ nanorods, Pt nanoparticles

## Abstract

Because the oxides of nitrogen (NO*_x_*) cause detrimental effects on not only the environment but humans, developing a high-performance NO_2_ gas sensor is a crucial issue for real-time monitoring. To this end, metal oxide semiconductors have been employed for sensor materials. Because in general, semiconductor-type gas sensors require a high working temperature, photoactivation has emerged as an alternative method for realizing the sensor working at room temperature. In this regard, titanium dioxide (TiO_2_) is a promising material for its photocatalytic ability with ultraviolet (UV) photonic energy. However, TiO_2_-based sensors inevitably encounter a problem of recombination of photogenerated electron-hole pairs, which occurs in a short time. To address this challenge, in this study, TiO_2_ nanorods (NRs) and Pt nanoparticles (NPs) under a UV-LED were used as an NO_2_ gas sensor to utilize the Schottky barrier formed at the TiO_2_-Pt junction, thereby capturing the photoactivated electrons by Pt NPs. The separation between the electron-hole pairs might be further enhanced by plasmonic effects. In addition, it is reported that annealing TiO_2_ NRs can achieve noteworthy improvements in sensing performance. Elucidation of the performance enhancement is suggested with the investigation of the X-ray diffraction patterns, which implies that the crystallinity was improved by the annealing process.

## 1. Introduction

The oxides of nitrogen (NO*_x_*), which belongs to one of the toxic gases [1,2], have drawn enormous attention from researchers who develop gas sensors over the past decades. Because NO*_x_* gases are harmful to the environment and poisonous to humans, it is required that NO*_x_* sensors have high, reliable performance for real-time monitoring [3]. In this regard, to improve sensor performance, many researchers have been utilizing semiconductor metal oxides [4]. These semiconductor-type gas sensors are widely known to have a low cost and simple completion [5]. Although various sensing types have been suggested [6,7,8,9,10], using semiconducting metal oxides has further potential to take advantage of the growing field of nanotechnology [11].

One of the possible enhancements made by employing nanotechnology is the improved capability to work at room temperature (RT) in combination with photonic energy. Gas sensors made of metal oxide semiconductors usually work at high temperature in the range of 200–500 °C, which is likely to cause drawbacks such as limited lifetime or sensitivity degradation [11]. To surmount these limitations, studies on RT gas sensors have flourished in recent years [12,13,14,15]. The RT gas sensors, using metal oxide semiconductors, utilize photoactivation, which is a viable alternative to thermal activation, thereby lowering the working temperature. Because various nanostructures have been found to further improve the sensor performance at RT, RT gas sensors with nanostructures that use photonic energy have been developed in many recent studies [16,17,18,19,20].

Another possible improvement is further performance enhancement made by loading noble metal nanoparticles (NPs) on photoactivated metal oxide semiconductors. Titanium dioxide (TiO_2_) decorated with the noble metal NPs, such as Pt [21,22], Au [23,24], Pd [25,26], or Ag [27,28], is a widely known example. The charge separation, by which the photocatalytic activity is remarkably improved, is one of the expected effects, although the exact mechanism has not been disclosed clearly [29]. TiO_2_ has attracted attention as a promising material for gas sensors due to its non-toxicity, inexpensiveness, stability, and photocatalytic ability [30]. However, there is an undesirable possibility that photoactivated electrons are not allowed to reach the surface for chemical reactions because the time for the recombination of the electron-hole pairs is too short. In this regard, to extend the time for the separation between the electron-hole pair, the noble metal NPs have been utilized. The noble metal NPs on TiO_2_ work as electron traps by forming the high Schottky barriers at the interface, thereby significantly improving the performance [21,22,29].

In this study, NO_2_ gas sensors made of annealed TiO_2_ nanorods (NRs) and Pt NPs are developed to work at RT by utilizing the photoactivation and charge separation. TiO_2_ NRs are fabricated by the well-known hydrothermal method [31] which has several advantages compared to the other fabrication methods [32], and subsequently, TiO_2_ NRs are annealed with the expectation that the heat treatment might cause changes in crystalline characteristics and photocatalytic properties [33]. The agglomeration of Pt using rapid thermal processing is utilized to load Pt NPs on TiO_2_ NRs. The as-prepared sensor is photoexcited to detect NO_2_ gas at RT by using an ultraviolet light-emitting diode (UV-LED). After setting an experimental setup, gas sensing performances are tested to investigate the effects of the annealing process. Herein, it is reported that annealing TiO_2_ NRs is likely to improve the performance of the RT gas sensor significantly owing to crystallinity improvement of TiO_2_ NRs by thermal treatment. Lastly in the paper, plausible photocatalytic oxidation mechanisms are proposed to explain the results of the experiments.

## 2. Materials and Methods

### 2.1. Fabrication Process

**Figure 1:**
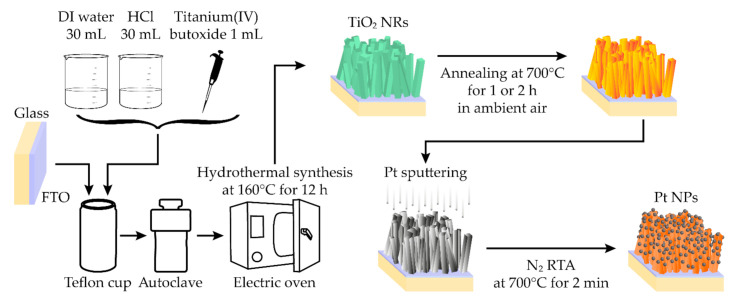
A schematic of the fabrication process is illustrated. The hydrothermal method is employed to obtain titanium dioxide (TiO_2_) nanorods (NRs) on the substrate of fluorine-doped tin oxide (FTO)-coated glass. After annealing the TiO_2_ NRs, the agglomeration of Pt is utilized by conducting rapid thermal annealing (RTA) to load Pt nanoparticles (NPs) on TiO_2_ NRs.

Figure 1 shows the process of fabricating TiO_2_ NRs loaded by Pt NPs. A fluorine-doped tin oxide (FTO) glass (14 Ω/square) was purchased from Pilkington (Japan). The FTO-coated glass was prepared after ultrasonically cleaning with EnSolv, acetone, isopropyl alcohol, and deionized (DI) water. To prepare hydrothermal precursor solution, hydrochloric acid (M = 36.46, HCl) and titanium(IV) butoxide (97%, TBO) were purchased from Daejung (Korea) and Sigma-Aldrich, respectively. 30 mL of DI water and 30 mL of HCl were mixed and stirred for 5 min. 1 mL of TBO was dropped into the solution of DI water and HCl, and the solution was stirred for 5 min. The as-prepared solution was poured into a Teflon cup and subsequently, the cleaned substrate was placed vertically in the Teflon cup. After sealing the Teflon cup with a stainless steel autoclave, the autoclave was tightened to prevent leakage of the solution at an elevated temperature. The hydrothermal synthesis was conducted at 160 °C for 12 h. After cooling the autoclave and its contents, synthesized TiO_2_ NRs were rinsed with DI water and dried with nitrogen gas. Subsequently, the TiO_2_ NRs were annealed at 700 °C in ambient air. Herein, annealing time was controlled to investigate the effects of heat treatment. To form Pt NPs on the surface of the TiO_2_ NRs, after sputtering Pt for 4 min by using the sputter coater (Cressington, 108 Sputter Coater), rapid thermal annealing (RTA) was carried out to Pt-sputtered TiO_2_ NRs at 700 °C for 2 min in nitrogen ambiance. Because the RTA leads to the agglomeration of Pt on TiO_2_ NRs, Pt-NP-loaded TiO_2_ NRs were obtained consequently. The abbreviations of the types of the samples used in this paper are listed in Table 1.

### 2.2. Characterization Method

The morphologies of the prepared samples were investigated by using the field-emission scanning electron microscope (FE-SEM, FEI Company, Nova NanoSEM 200) at 10 kV. The X-ray diffraction (XRD) patterns were measured to examine the crystal phase by using the X-ray diffractometer (Rigaku Corporation, SmartLab). The measurements of XRD patterns were performed with the copper target (λ = 1.540562 Å) at 9 kW and in the range of 20° to 70° at a speed of 5°/min.

### 2.3. Experimental Setup for NO_2_ Gas Sensors

**Figure 2:**
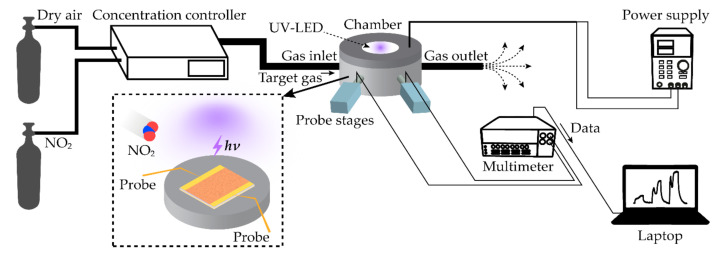
An experimental configuration of measuring gas sensing properties is depicted. The concentration controller is a customized system which controls concentrations of the target gas. The inset shows a schematic diagram describing the test chamber. The UV-LED is attached to the top surface. The sample of the gas sensor is laid on the bottom. On the laptop, resistances of the sample obtained by the multimeter are plotted in real-time.

Figure 2 depicts a schematic of an experimental setup where the as-prepared samples were tested for sensing NO_2_. Dry air and NO_2_ gas flowed into a customized controller to obtain concentrations, such as 5 ppm, 10 ppm, or 15 ppm. Mass flow controllers and air drive forming machines were deployed in the controller, and the control system was manipulated by using LabVIEW software. The 500 sccm of target gas of which concentration was controlled filled a test chamber and subsequently, flowed out through the outlet. The inset in Figure 2 shows a schematic inside the test chamber. The UV-LED of which wavelength is 370 nm was located on the top inside the chamber and driven by the power supply (MKPOWER, MK3005D, at 20 mA) which was placed outside of the chamber. The UV-LED remained on during the test experiments. The sample of the gas sensor, of which dimensions were 2 cm × 2 cm, was laid on the center of the bottom. The positions of the probes were adjusted with the stages connected through the wall of the chamber. Electrical signals from two probes were sent to the multimeter (Keithley, 2700 Multimeter). Lastly, the data of the resistance was plotted on the laptop. Every experiment was conducted at RT around 20 °C.

## 3. Results and Discussion

### 3.1. Characterization

**Figure 3:**
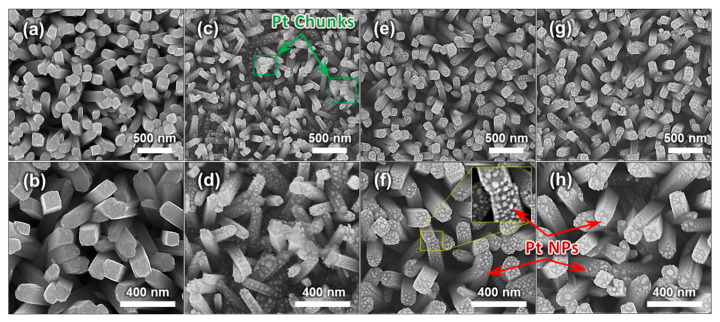
FE-SEM images of the gas sensors are shown. (**a**,**b**) are the results of the hydrothermal synthesis. (**c**,**d**) are TiO_2_ NRs and Pt NPs of Sensor A. (**e**,**f**) are TiO_2_ NRs and Pt NPs of Sensor B. (**g**,**h**) are TiO_2_ NRs and Pt NPs of Sensor C. The inset in (**f**) shows an enlarged image to clearly display the Pt NPs on the TiO_2_ NRs. The red arrows indicate Pt NPs. The green arrows in (**c**) indicate chunks of Pt agglomerated together on the top surface of TiO_2_ NRs.

Figure 3 shows the morphology of the samples fabricated following the process depicted in Figure 1. It is confirmed that TiO_2_ NRs were grown evenly. The average density of the number is obtained as 36/μm^2^ approximately by counting the NRs in the FE-SEM images of Figure 3. There are adequate sites for the target gas to be adsorbed on the surface, thereby probably increasing the gas sensing performance [22]. Figure 3a–d present the results before and after loading Pt NPs, respectively. TiO_2_ NRs in Figure 3e–h were annealed for 1 h and 2 h, respectively, and decorated with Pt NPs. Namely, Figure 3c–h are the FE-SEM images of Sensor A, Sensor B, and Sensor C, respectively. Based on the FE-SEM images of Figure 3, the TiO_2_ NRs have an average diameter of about 83 nm with a standard deviation of around 21 nm. Whereas the size of the Pt NPs in Figure 3c,d is around 13–42 nm, the Pt NPs in Figure 3e,h have the size of 13–18 nm approximately. In addition, as shown in Figure 3c,d, for the TiO_2_ NRs which did not have an annealing process, the Pt NPs on the top surface agglomerated together to form a chunk of Pt. Lastly, cross-junctions between NRs contacting each other which offer pathways for electrons are observed [34].

**Figure 4:**
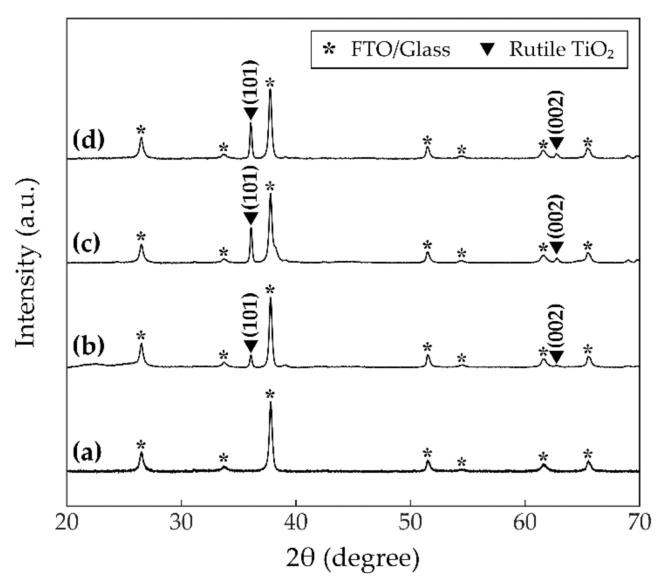
X-ray diffraction (XRD) patterns of (**a**) the FTO-coated glass as the substrate, (**b**) Sensor A, (**c**) Sensor B, and (**d**) Sensor C. The asterisk denotes the peak of the substrate and the triangle marker indicates the peak of the rutile TiO_2_.

The XRD patterns are shown in Figure 4 to investigate the crystallinity of the TiO_2_ NRs. Figure 4a shows the XRD pattern of the substrate before synthesizing TiO_2_ NRs, and the peaks are denoted by the asterisks. The XRD pattern of Sensor A is plotted in Figure 4b. In this case, TiO_2_ NRs did not have an annealing process before loading Pt NPs. Compared to Figure 4a, two peaks emerge at 36.1° and 62.7° which correspond respectively to the rutile (101) and (002) peaks referred by JCPDS card no. 21-1276. Because the hydrothermal method used in this study initiates the (101) facet more easily, it can be noticed that the peak of the (101) plane is dominant [35]. Although anatase, one of the possible crystalline forms of TiO_2_, is the most famous for photocatalytic activities, the rutile phase could be desirable for photocatalytic oxidation activities [36]. Figure 4c,d plot the XRD patterns of Sensor B and Sensor C, respectively. In all three cases of Figure 4b–d, the peaks of Pt do not appear. This unobservable characteristic implies that the fine Pt NPs are highly dispersed on the surfaces of TiO_2_ NRs [37].

**Figure 5:**
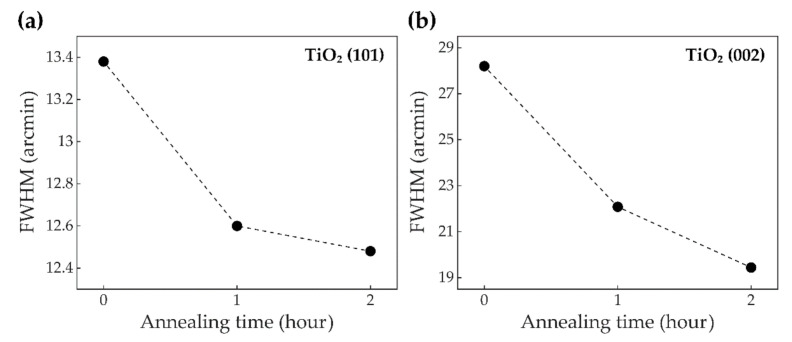
The full width at half maximums (FWHMs) of the XRD peaks of the rutile TiO_2_ with respect to time of annealing TiO_2_ are shown. (**a**) and (**b**) are the results at the (101) peaks and (002) peaks in Figure 4, respectively.

Figure 5 implies that annealing TiO_2_ NRs in ambient air is likely to improve crystalline characteristics of the TiO_2_ NRs [38]. The full width at half maximum (FWHM) is calculated from the XRD patterns obtained in Figure 4. Figure 5a,b show FWHMs of Figure 4b–d at rutile TiO_2_ (101) and (002) planes, respectively, with respect to time of annealing TiO_2_. For the (101) facet, annealing processes for 1 h and 2 h give decrements of 5.8% and 6.7% on the FWHMs relative to the FWHM of the sample without thermal treatment. Likewise, for the (002) peak, the FWHMs after annealing 1 h and 2 h are decreased by 21.7% and 31.1%, respectively. These decremental FWHMs with respect to time of annealing TiO_2_ indicate incremental improvements to the crystallinity of TiO_2_ NRs. It is expected to lead to enhancing electron mobility, thereby inhibiting electron-hole recombination. This suppressing recombination will offer photoactivated electrons the strong possibility to reach the surface of the TiO_2_ NR, accumulate in the Pt NPs, and subsequently, provide a photoelectrochemical response.

### 3.2. Sensor Performance

**Figure 6:**
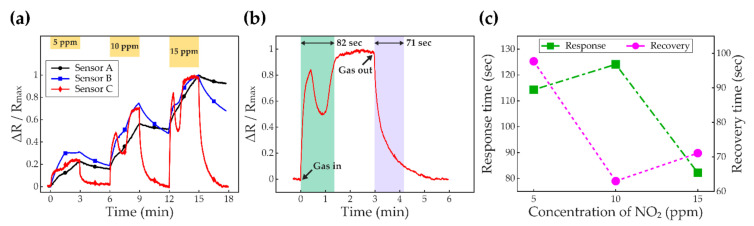
Sensor performances are plotted. (**a**) shows the effects of annealing on performance. The yellow bar graph on the top of (**a**) depicts the concentration of NO_2_ with respect to time. The black line marked by the circles shows the response of Sensor A. The blue line labeled by the squares and red line marked by the diamonds plot the responses of Sensor B and Sensor C, respectively. For the *y*-axis, the resistance increment is normalized with respect to the maximum value of each graph to compare the curve profiles of three sensor types. (**b**) depicts the response of Sensor C when the sensor is exposed to the 15-ppm NO_2_ gas. The response time and recovery time are denoted in (**b**) as 82 s and 71 s, respectively. The moments of gas in and out are indicated by the black arrows. (**c**) shows the response time and recovery time of Sensor C. Left and right *y*-axes indicate the response time and recovery time, respectively. The green square and magenta circle denote the response time and recovery time, respectively.

Figure 6 shows the NO_2_ sensing performances at RT with the UV-LED. The experiments depicted in Figure 2 were conducted for three cases of the samples; Sensor A, Sensor B, and Sensor C. When the sensors were deployed without UV exposure, the read-outs showed only noisy signals. Figure 6a compares the results of the experiments obtained from each sample when the concentrations of the NO_2_ gas were controlled to be 5 ppm, 10 ppm, and 15 ppm. For comparisons of the performance curve profiles of three sensor types, the resistance increments, relative to the value of the baseline, were normalized with respect to the maximum value of each case; that is, ΔR/R_max_. The baselines were obtained by waiting for 30 min after turning on the UV-LED to stabilize the sensors. The time interval between gas on and off was set to be 3 min. When the sensors are exposed to the NO_2_ gas, the resistances increase as electron depletion layers become wider. In addition, the increments in the resistances are proportional to the concentration of the NO_2_ gas. Especially, Sensor C has saturated values of the resistance. As shown in Figure 6a, for 5 ppm, 10 ppm, and 15 ppm NO_2_ gas exposures, the saturated values in the *y*-axis of Sensor C are obtained as 25%, 70%, and 100%, respectively. For 10 ppm and 15 ppm of the NO_2_ gas, it is observed that there are overshoot characteristics, and the overshoot becomes sharper as the concentration of the NO_2_ gas increases. Although the overshoots occur, the response becomes steady and saturated eventually. Moreover, the sensor makes a complete recovery within 3 min after turning off the NO_2_ gas. However, Sensor A shows a slow response so that the resistance value does not saturate. Besides, in this case, because the recovery time drags, the sensor fails to make a full recovery. For Sensor B, the rates of the response and recovery are noticeably improved compared to the sensor without annealing. Nevertheless, it is evident that further improvements are still required. Figure 6b enlarges the sensing performance of Sensor C when the sensor was exposed to 15 ppm of the NO_2_ gas as a representative. Although there was an overshoot when resistance was increasing, the saturation property was confirmed. The response time, the time to arrive at 90% of the saturated value, is obtained as 82 s. When turning off the NO_2_ gas, the curve makes a complete recovery, and the recovery time—the time to recover 90% from the saturated value to the baseline—is identified as 71 s. Figure 6c depicts the response time and recovery time of Sensor C according to the concentration of the NO_2_ gas. When the sensor is exposed to the 10-ppm NO_2_, compared to the 5-ppm NO_2_, the recovery time shortens, whereas the response takes a longer time because the saturated value is reached after passing through the overshoot characteristic. In contrast, for the higher concentration of the NO_2_ gas, 15 ppm, response time decreases, whereas the recovery time becomes extended.

### 3.3. Discussion on Sensor Mechanism

Herein, the NO_2_ sensing mechanism by TiO_2_ NRs and Pt NPs is proposed. Because the contact between TiO_2_ and Pt is a metal-semiconductor junction, a Schottky barrier is formed [22]. Illuminating UV photons, the charge separation occurs, possibly due to changes in the surface potential [29]. In this regard, by attaching Pt NPs to TiO_2_ NRs, Pt can capture the photogenerated electrons from TiO_2_. The electron separated from the electron-hole pair reacts with NO_2_ gas at the surface, and subsequently, it leads to intensifying the electron depletion layer of TiO_2_, which is the n-type metal oxide semiconductor [39]. It was confirmed that in Figure 6, the resistance of the sensor increases when the sensors are exposed to NO_2_ gas.

However, it is known that the photoexcited electrons, traveling in TiO_2_, are barely able to reach the surface of TiO_2_, which includes the metal-semiconductor junction, when the electron-hole recombination occurs too rapidly [29]. To address this issue, the electron mobility in TiO_2_ needs to be fast enough for electrons to pass over from TiO_2_ to Pt. As demonstrated in Figure 5, annealing TiO_2_ NRs in the fabrication process can significantly improve the crystallinity of the TiO_2_ NRs [38]. These annealing processes were conducted at 700 °C in ambient air for 1 h and 2 h after hydrothermally synthesizing TiO_2_ NRs as depicted in Figure 1. The improvements in crystallinity quicken the movements of the photogenerated electrons.

**Figure 7:**
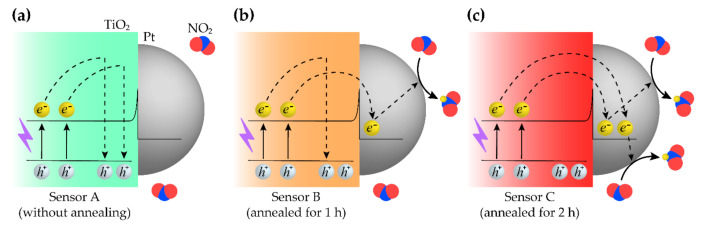
Proposed sensing mechanisms are depicted. Photoelectrochemical activities for sensing NO_2_ with respect to time of annealing TiO_2_ are shown. (**a**) is the schematic of Sensor A which did not have an annealing process to TiO_2_. (**b**) and (**c**) suggest the working mechanisms of Sensor B and Sensor C of which TiO_2_ NRs were annealed for 1 h and 2 h, respectively. e^−^ and h^+^ indicate electron and hole, respectively.

Figure 7 illustrates the proposed mechanism described above. Figure 7a is a schematic of Sensor A. As shown in Figure 6a, this type of sensor had poor performance for the too slow response. It is believed that too many electrons are recombined with the holes, and these electrons cannot be involved in the surface reactions. In contrast, Figure 7b,c depict Sensor B and Sensor C, which have the annealed TiO_2_ NRs with the improved crystalline characteristics. However, for Sensor B, further improvements in the crystallinity are still required. Further enhancement can be achieved by annealing TiO_2_ NRs for 2 h. As demonstrated in Figure 5, Sensor C had better crystallinity than Sensor B. Therefore, the photogenerated electrons run swiftly to reach the interface between TiO_2_ and Pt before recombinations of the electron-hole pairs. The Pt NPs hold the electrons like electron traps by utilizing the Schottky barriers [21,40]. Finally, it leads to the fast sensing response, as demonstrated in Figure 6, by letting the electrons react with the NO_2_ gas at the surface.

Because for TiO_2_-Pt, higher crystallinity might yield higher photocatalytic activity at room temperature [41,42], there is a possibility that improvement in crystallinity could lead to enhanced photocatalytic activity. Presumably, there is another chance that wider bandgap with higher crystallinity could strengthen the effects of the Schottky barrier, thereby enhancing the charge separation [43].

As indicated in Figure 3c, for Pt NPs and non-annealed TiO_2_ NRs, it was noticeable that Pt NPs clumped together to form a chunk of Pt on several locations. Because Pt on these sites might capture holes as well as electrons, the electrons eventually come to recombine with the holes [40]. Consequently, it could contribute to the poor sensing performance of Sensor A. Meanwhile, the UV light illumination induces plasmonic effects in the fine Pt NPs. Because the plasmonic effects can inhibit electron-hole pair recombinations, photoelectrochemical activities are probably enhanced [22]. This improvement is likely to contribute to the faster response of the sensor.

It needs to be noted that a reproducibility study, which is especially important for on-field applications [44], is further required to ensure robust sensor performance. Because humidity probably affects the electron mechanism [34,40], humidity control should be covered in the reproducibility study.

## 4. Conclusions

In this paper, TiO_2_ NRs and Pt NPs under UV light irradiation have been proposed for the RT gas sensor. TiO_2_ NRs were prepared by the hydrothermal synthesis, and the as-prepared TiO_2_ NRs were annealed in ambient air. It was demonstrated that by analyzing the XRD patterns, the 2-h-annealed TiO_2_ NRs had significantly improved crystallinity compared to the non-annealed TiO_2_ NRs. It can lead to the faster movements of the photoactivated electrons and suppressing electron-hole recombinations. Pt NPs were fabricated by sputtering Pt and subsequently, performing RTA in N_2_ ambiance. FE-SEM images showed that the agglomeration of Pt occurred, and TiO_2_ NRs were successfully decorated with Pt NPs. By utilizing the Schottky barrier between the Pt-TiO_2_ junction, the photogenerated electrons can be captured by Pt and exposed to NO_2_ gas. The plasmonic effects by the fine Pt NPs are believed to further accelerate the separation between the electron-hole pairs. To evaluate NO_2_ gas sensing performance, the experimental platform was set up. In the experiments, the values of resistance of the gas sensor were measured in real-time. It was confirmed that the sensing performance of the sensor with the 2-h-annealed TiO_2_ NRs was greatly superior to the performance of the sensor with the 1-h-annealed or non-annealed TiO_2_ NRs. It suggests that improving the crystallinity by carrying out thermal treatment is a highly effective method for the photoactivated electrons to reach the surface. Therefore, the 2-h-annealed TiO_2_ NRs decorated with the plasmonic Pt NPs have been employed for RT detection of NO_2_ gas successfully by using UV photonic energy.

## Figures and Tables

**Table 1:** Abbreviations of the sample types are listed. The samples were fabricated by following the process illustrated in Figure 1.

Abbreviations for Sensor Types	Time for Annealing TiO_2_ NRs at 700 °C
Sensor A	No annealing
Sensor B	1 h
Sensor C	2 h
